# On Hallucinations
*"Des Hallucinations; ou, Histoire Raisonnée des Apparitions, des Visions, des Songes, de l'Extase du Magnétisme et du Somnambulisme." Par A. Brierre de Boismont, Docteur en Médecine de la Faculté de Paris, &c. Seconde Edition. Paris, 1852.


**Published:** 1853-04-01

**Authors:** 


### Art. II.—ON HALLUCINATIONS.*

We did not open this voluminous treatise of Monsieur de Boismont without being fully prepared for much learning and research; not, however, without a certain degree of wonder, that even an able physician could find in these illusions of the sense or senses, which have usually been deemed concomitants or even mere symptoms of deeper maladies, matter enough for more than 700 pages! and not without a fear, also, that we must inevitably be crossed by cases and records which to us, or by us have been already ten times repeated.

Every one must confess the deep importance of the subject. There is none *more so*, as it intimately concerns the mysteries of the immortal spirit, and the health of the body, as well as the earthly happiness of mankind.

Now, however varied, and however evident and conclusive the *exciting* causes of hallucinations may be, the great question of the *proximate* cause of illusion, and of the various forms of insanity, is still *sub judice.* And we fear that, after the most protracted discussion between the spiritualist and the materialist, or the pathological psychologist and the metaphysician, the subject would still be left in *statu quo.*

The perusal of the second page forewarns us of the psychological creed of the author; and the student must be prepared to study the book with some caution, regarding the portion of abstract metaphysics, in reference to the manifestations of mind. The pure spiritualist cannot for a moment assert his creed without at once admitting the existence of *disorder* in that immortal essence of our being, which is the pure emanation of the Deity pervading all things. The abstract materialist, on the contrary, is engaged in an incessant search for *evidence* of some morbid condition or change in that organ, which is

* "Des Hallucinations; ou, Histoire Raisonnée des Apparitions, des Visions, des Songes, de l'Extase du Magnétisme et du Somnambulisme." Par A. Brierre de Boismont, Docteur en Médecine de la Faculté de Paris, &c. Seconde Edition. Paris, 1852.

the habitat of the immortal spirit during its earthly existence. Although these are the extremes of psychological theories, they are not, however, equidistant from the truth.

We know that the Spirit of the Creator may, and does, pervade every atom of his beautiful and wondrous creation: but to make us sensible even of this sublime truth, to qualify us for perceiving, combining, and comparing our perceptions and our consequent thoughts, we possess a brain through which these processes must inevitably pass; we may hence assume that the phreno-psychologist is *nearest* the truth. *His* error often lies in the reluctance to acknowledge that of which he has not evidence, as if scores of transient morbid actions, and even of minute changes, were not constantly going on in the body, of which, probably, the most accomplished histologist will never be able to demonstrate the proof.

The circulation of the blood quickens by a thought or emotion, may be restored as soon as the thought has passed from the mind, and so may also thrills, rigors, flushes, and other evidences of emotion. We do not here pause to discuss the relative importance of nerve and blood in the causation of these phenomena; we have already fully commented on this point. No one will doubt that a certain degree of transient hallucination *may* take place in a state of bodily health and mental sanity—*so* transient as not to merit the term disorder—yet we cannot deny that every symptom which is abnormal or unnatural, may, nay must, often be the precursors of disorder. The very derivation of the terms hallucination and illusion imply this—that the one signifies a blundering or mistaking, the other the mockery of a false appearance.

We take, therefore, with some reservation this early passage in Dr. de Boismont's preface, which we suppose to be the creed or theme of his dissertation.

"The first design of this book is clearly established. We wish to protest against the hypothesis that hallucination is a constant symptom of derangement—in certain cases it may be a phenomenon purely *physiological.*"

Now, we have ever taken the science of physiology as the *laws* of the animal economy. To see without an eye—to neutralize, as it were, the functions of the senses, must be to abrogate such laws, and this, being therefore abnormal, it can scarcely be considered as within the pale of physiology.

The subject of the book being therefore confessedly *false perceptions*, our own position will be at once evident.

The proposition, however, that hallucination is not necessarily a symptom of real *insanity* is quite another question, requiring, indeed, a very patient and serious discussion. We therefore leap over the preface and introduction, that we may devote all our comments to a running analysis of this learned work.

If there be any point in our science more vague and more conducive to misapprehension, it is *definition:* of this the discrepancies which Mons. de Boismont displays in his quotations from Aristotle to our own time are a proof. Yet, although these definitions, even from Arnold to Esquirol, so differ among themselves, we can by analysis trace, even in this difference, the same trains of thought which led to the conclusion—the mere terms often constituting the difference. To attempt fully to analyze and collate these discrepancies would argue, we fear, a sort of *folie raisonnante* in our own persons, especially if we endeavour to bring them together—to show why some deem hallucination merely spiritual, others as "lesions des organes des sens:" to try to convince of their errors, others who jumble and associate with hallucination optical and atmospheric spectra which have no reference whatever to the state of the brain or mind, being mechanical or substantial; to decide, in short, which is right: whether Esquirol, who affirms hallucination to be essentially *idiopathic,* or Calmeil, who believes it to be constantly *symptomatic.* Why, we should, after all this, assuredly become apt illustrations of the subject we are discussing.

We believe that the clearest definition of phantasma would be, "illusive conception the creation of phantoms from former impressions, and illusive perception the conversion of natural objects into phantoms." We quote from Mr. Dendy's "Philosophy of Mystery" rather to explain what Mons. Boismont has rather vaguely translated into "souvenir passé, and souvenir présent." The whole tenour of that work, regarding our present subject, we may add, closely coincides with our author's later definition—"L'hallucination, la perception des signes sensiblés de l'idée: et l'illusion l'appreciation faussé de sensations réelles."

Believing, then, that the spring of ideas is the memory of impression, we of course do not coincide with the doctrine of their *innate* essence. With this reservation, we recommend our author's table of classification (page 28) to the careful perusal of the psychologist. It is, of course, the basis of his treatise.

The anxiety of M. de Boismont is to establish the physiological nature of certain hallucinations: the paramount proposition in the book, therefore, is the compatibility of illusions of the mind and senses with sanity, or judgment ("la raison"). We see at once what the author means by the proposition; and he *may,* of course, *be* right; but the notion is one of much greater delicacy and difficulty than *primâ facie* may be evident; and we dare to say it will be widely objected to, if not controverted. No one will hesitate, *in limine,* to concede, that hallucination may suddenly or gradually display itself in minds of the greatest energy and intellectual superiority; indeed, it is these choice spirits, especially when extreme energy or excess of work has overstrained the mental organ, that phantasy is more likely to invade, than beings of a lower intellectual capacity. It is clear, then, that hallucination is not essentially a symptom of confirmed or general insanity, as the term is conventionally understood.

But to argue that the mind, or rather its organ the brain, is at that moment in a perfectly sane or physiological condition—*i. e.*, during the paroxysm, is quite another thing. Hallucination must often at that time come under the definition of monomania: to strain somewhat John Hunter's physical axiom, psychologically, a mind cannot be sane and insane at the same moment, however we may be disposed to coincide with our late worthy friend Dr. Wigan, on his ingenious principle of duality.

This illusion may be transient, it may be momentary, depending, indeed, on the state of the current of the blood; but we fear we are anticipating, as we pen these lines, before we have commenced our author's first chapter, in which, ere he cites the facts and anecdotes recorded by psychological writers, he first alludes to the distinctions and analogies of reason and insanity, between which, as indeed we have often before stated, there may be but a hair line of demarcation.

In reading further we feel more disposed to press the dissociation of spectral illusion from ocular spectra, illusions of the mind from those of the eye; this established, it might indeed render the physiological and pathological division almost needless. All must believe, with Newton, that we can create phantoms on optical principles; or, that there are illusions of the senses, mysteries in art and nature, which we cannot fully explain; these, of course, are not pathological hallucinations, but come rather under the head of natural magic than hallucination.

The state of healthy *reverie*,—*i. e.*, the concentrated working of intellectual thought, is, of course, physiological; but the author has been confused with the illusions of deuteroscopia, or second-sight. The records of Martin the historian of the Hebrides, of Walter Scott, and others, prove at once the morbid state of mind of the seers; and this is the description of Collins, in his Ode on Highland Superstition.

> "They whose sight such dreary dreams engross,
> With their own vision oft astonished droop,
> When, o'er the wat'ry strath or quaggy moss,
> They see the gliding ghosts embodied troop.
> They know what spirit brews the stormful day,
> And heartless oft, like moody madness, stare
> To see the phantom train their secret work prepare."

Closely allied to deuteroscopia is the fine frenzy of the poet, on which so often comes in the end despondency and madness. Indeed our author is on or close to the pathological line when he pens this sentence. Of a truth, its labyrinth is perilous to those who possess not an eye sure enough, or a foot sufficiently firm, to keep them in the right track!

Some poets and philosophers of course prove this abstraction a leading star. "For St. Jean Chrysostom, for Descartes, Malebranche, Dante, Milton, Spinosa (Shakspere, who soared immeasurably beyond all in the æther of imagination, is omitted!) *reverie* is force, power, health, and often indeed longevity. To them solitude is a blessing." On this principle we quote the following remarks of Meister from our author's book. If he adopt the term reverie, we agree; if that of illusion, we differ.

"I am persuaded that devotees, lovers, prophets, *illuminés*, Swedenborgians, are indebted to illusions for all the wonders of their presentiments, their visions, their prophecies, their intercourse with celestial beings, their excursions to heaven and hell,—in a word, all the extravagancies, all the superstition of their infectious reveries. But I will also fearlessly affirm that, *under just such a state* men of genius have conceived the most original beauties of their compositions, that the geometrician has solved the problem which has long puzzled his brain, the metaphysician hit on the most ingenious of his theories, the poet on the line that had long escaped him, the musician the most brilliant and expressive of his movements, the statesman developed the decisive remedy which all his previous calculations, aided by the light of experience, had failed to do, the general attained that comprehensive *coup d'œil* that decided the fate of battle and ensured him the victory."

The influence of climate and locality cannot be doubted. M. de Boismont writes—"Those who have lived in eastern climates, or who have written on their beauty, all assert their powerful influence in exalting the imagination of their inhabitants." The author proceeds to detail the contrasted habits and diet of the orientals and the western people, concluding with this sentence—"It is to this power of their imagination that we owe the wondrous oriental tales; it is that which peoples the interior of the globe with genii, magicians, and enchanted palaces," &c. &c. We might go on to argue on the proximate cause or cerebral condition which divide the physiology from the pathology of this question; but that would smack of repetition, and we content ourselves by merely offering the brief psychical explanation, that in the one case the judgment waits upon the train of thought; in the other, imagination steals the thought, and converts the workings of reason into a mere flight of fancy.

The truth is, the mind may be *generally* rational during hallucination. M. de. Boismont alludes to artists, who at one sitting have so deeply impressed the brain with form and expression, that they could at will call up the eidolon of the sitter. And, among other analogous stories, he has recorded the following anecdote: "Hyacinthe Langlois, a distinguished artist of Rouen, an intimate friend of Talma, told us that the great actor confessed to him, that when he was on the stage, he possessed the voluntary power to unrobe (disparaître les vêtements de) his elegant and numerous auditory, and to substitute for these living beings a set of skeletons." Something of this kind often affected Mrs. Siddons after playing Lady Macbeth. The one was voluntary, however, the other involuntary; yet we may fain ask whether the proximate cause, the condition of the brain, was not probably the same in both. The bloody head of Count D'Olivarez, the great cat case related by Wigan, the story of the illustrious patient of the Demonology, the cases of Madame A. by Brewster, of Nicolai, of Berlin, and others, of which we are almost tired of thinking, must come under the same category. The psychical difference may be, that some *retain*, while others *want* the *comparing* faculty. That the influence of position and the circulation of the blood must often form a most prominent point in discussion, is at once displayed by the case which M. de Boismont quotes from Dendy's "Philosophy of Mystery" (which contains, however, still more prominent cases):—"A gentleman of high attainments was constantly haunted by a spectre when he retired to rest, which seemed to attempt his life. When he raised himself in bed, *the phantom vanished, but reappeared as he resumed the recumbent position!*"

In the second subsection, page 59, many very interesting cases are recorded; but are these cases, uninfluenced by judgment, (non rectifées par l'entendement) to be considered as "compatibles avec la raison?" There must be derangement of intellect here. The well known case of the late Lord Londonderry is recorded. It is clear all this was pathological. His lordship (whom, with a touch of amor patriæ, M. de Boismont calls "le plus acharné persécuteur de Napoleon dans son malheur") was labouring under "un tour melancholique aux pensées" when the first illusion of the *radiant boy* came before him in the very chamber of the old castle in Erin; and the second apparition, in the House of Commons, there is no difficulty in explaining by anxiety and cerebral congestion. The stories of the Baron Geramb at Cadiz, and the haunted house of Athenodorus at Athens, which are quoted from the "Philosophy of Mystery," as well as all other specimens of the Poltergeist especially, cannot be termed illusions any more than the tricks at Woodstock, the Cock-lane and Stockwell ghosts, and a host of others; they are mere ghost stories. The two interesting anecdotes quoted from the able works of Mr. Dendy and Dr. Wigan, in which a vision appeared *synchronously* to more than one, call forth the following remarks: Does not the concluding word *divination* at once beg the question, and draw the learned author away from the truth of this deep and difficult question?

"These illusions may be to a certain point explained by the sympathetic ties (liens) between the members of attached families, and by the psychological resemblance that has often struck us, especially between husband and wife, so that the thoughts seem to pass from one to the other without any actual communication, but by a *species of divination.*"

Now is this aught else than coincidence?

The hallucination of a sense, when its organ has totally lost its faculty, is, *prima fácie,* almost a mystery; the electro-biologist pretends to confer it at his will. One of the most curious cases is quoted from Calmeil, of the *deaf* ecclesiastic. They are, however, merely instances of excited memory, analogous indeed to the ghost of the amputated limb. After long dwelling on one point, how often is the eidolon raised up, subjectively or objectively. Hallucinations of the smell and taste are generally combined with illusions of the other senses.

In his résumé, p. 117, Mons. Boismont hazards this opinion,—"general hallucination may be a powerful argument in favour of *Berkleisme, if* a pathological state could prove a physiological principle." Much virtue in this *if.*

The definition, by Esquirol, of hallucination and illusion, described in page 119, is but, in other words, illusive perception and illusive conception, to which we have before often alluded.

We almost regret that M. de Boismont should have introduced the Brocken, and Morgana, as they merely tend to swell the volume, and, indeed, are destroying its perspicuity and concentration. They belong to Brewster, and not to Boismont. On the subject of epidemic or contagious hallucination, our author offers very just remarks. "Independently of the reasons we have given, especially those of ignorance, fear, superstition, and disease, we must not forget the influence of example. One exclamation (cri) will affright a multitude of people. He who believed himself the subject of supernatural visitation, might easily convince those who could not see more clearly than himself." The Spectre Hound, or Manthe Dog, of Man, is one of the most interesting anecdotes illustrative of this point.

It is of great importance that we should not disregard *slight* illusions; as Mons. de Boismont observes, "the visionary may at first be conscious that they are *false* perceptions; but, the malady increasing, he at length believes them *real.*" The case of the Blanchisseuse, p. 130, is an apt illustration. Protracted illusion, indeed, often induces melancholy and its train of evils. Hallucination is often productive of very important consequences, proving what great events may spring from little things. The vision of a lover, in a state of abstract reverie, may leave so deep an impression, that it may irresistibly impel him at once to the confirmation of his passion: and Mons. de Boismont relates, p. 133, a very interesting case of a gentleman who was constantly attacking his friends and his wife, from a powerful conviction that they were all demons by which he was beset. All the cases of this kind which we have known or read of, are explained on the principles of concentrated impression, or of impetus, or inducement.

Regarding the combination of hallucination with monomania, the calculation of Esquirol was about 80 per cent.; that of Mon. Baudry, in the Bicetre, about 70 per cent.; that of Aubanel about 50. Mons. de Boismont thinks Esquirol exceeds the number, but we believe so great a majority of these monomaniacal illusions to be *objective*, whether we term it eidolon or idea, that we are convinced Esquirol is right. The tables in p. 141, on this point are curious: the most frequent illusions are lypomania, nostalgia, demonomania, and erotomania.

On the principle to which we have alluded, we believe, with the author, that hallucination, when pathological, affects in preference the monomaniacal form; it may be, in fact, slight monomania, but we do not think the most common illusion to be that of the organ of hearing —rather that of sight.

That melancholy (*stupidité*) is a frequent source of hallucination, must be expected; and Mons. de Boismont affirms, that "in almost every example the conduct and eccentricities of the patients were evidently springing from hallucination."

In the hallucination associated with general insanity, vision is certainly the sense most subject to the mockery. The author says that in this form the combination of hallucination and illusion is most common, and they are constantly changing from one to the other. They do not seem, however, always to terminate with the mania. They are sometimes the very source itself of the derangement, at others merely one symptom of it.

One of the most interesting cases in the book is that of Mdlle. O. (page 175), a lady of great judgment, who often, in the midst of the most incoherent chattering, wrote letters full of sensible remarks, and without a word by which her illusion might be discovered. In a state of imbecility (démence), hallucination is also frequent. In complete idiocy, of course, there can be none. In the imbecile paralytic, illusions of the sight and sound are often combined, and these are often more protracted as the disease is more chronic and permanent. Among the cases of delirium tremens, in Charenton, the proportion of men being four times that of women, hallucination was very frequent, and seemed to indicate or discover the former habits of the patient.

Mons. de Boismont has not often found hallucination in cases of catalepsy, epilepsy, &c.; insensibility is so constantly present, that the mind is a tabula rasa. Previous to the attack, however, they are frequent, and the illusion assumes the form usually of cheromania, like Benvenuto Cellini, which is so well known. "I have among my patients," writes Mons. de Boismont, "a gentleman, who just previous to losing his consciousness had the most beautiful landscapes passing before his eyes."

When demonomania is the form it so marks the expression and contortions of epileptics, during the fit, that these seem, according to Esquirol, to be not only the result of, but characterised by, the horrid phantasy.

In the solitary form of chorea, hallucination is seldom seen: with hypochondriasis, and hysteria, it is more frequent, probably arising from the extreme depression and consequent blood congestion.

"Madame C. at the approach of her hysterical attacks became timid and frightened; sometimes to so great a degree as to cry out for help. This extreme terror is caused by the most horrible figures, which make grimaces and threaten to beat her."

This hysterical hallucination will often be epidemic or imitative—witness the convulsionaries—the story of the nuns, &c., &c. On this point we would refer to the anecdotes related by the author, and the chapter on Imitative Monomania in the "Philosophy of Mystery."

Mons. de Boismont very justly observes, "nightmare presents many analogies with insanity; it is not therefore surprising that it is complicated with hallucination." We coincide with this, having long considered the dream itself to be a transient derangement. The spectres of incubus are probably always distressing—the "black dog" of childhood is almost proverbial. The phantasy usually disappears on awaking from the incubus, "but sometimes it continues during waking moments, and is believed to be reality." This state must not, however, be associated, as the author is inclined to do, with reverie. It is clear that it is this concentration of reverie and not incubus or dream to which Voltaire thus alludes, p. 232.

"Voltaire thought one day that he had *dreamed* the first canto of the Henriade. 'I said in my dream what I could scarcely have said when awake. I had then deep reflections in spite of myself, and without the least effort. Will or liberty I had not, and yet I associated my ideas with sagacity and genius.'"

So also we may consider Sir Walter Scott's allusion when he was puzzled on a subject, "Ah, we shall have it in the morning;" and the cases of Condorcet, Mackenzie, &c., and that also of Sir Isaac Newton, who was ever in a state of reverie, or, as he said, always "thinking unto it." All this is reflective energy, and must wait on the concentrated judgment.

But we come now to those phenomena which, although their manifestations may be physiological, are ever marked by pathological effects. It is true that, during ecstasy, magnetism, somnambulism, extraordinary mental phenomena, and laborious works are witnessed; yet they are constantly attended by morbid signs—and even as Monsieur de Boismont allows—"a state *painful* to the body."

In addition to the stories of Swedenborg, Boehm, Santa Teresa, &c. &c. that of Maria de Mœrl, p. 279, is very long and very interesting. But it is very true, as M. de Boismont affirms, that "the misfortune of magnetism and somnambulism is, that they have been displayed by charlatans and impostors, who have, as it were, ousted the scientific psychologist. Their exaggerations have been fatally detrimental to an *analysis* of the phenomena." And we may venture to add, that if the sceptics, as well as the proselytes, would back out of the field, the psychologist would then be induced to render the true *explanation* as clear as noon day.

We believe our author rejects, with us, the notion of prophecy (prevision) being aught but casualty; and we think Ferriar, Hibbert, and others agree with Abercrombie, that these hallucinations are often but the memory of a forgotten dream.

Somnambulism is thus well defined:—"The mind, as it is in a dream, is concentrated on its own peculiar impressions, which it takes for so many real external sensations; but the organs, being more obedient to the power of the will, the individual acts and speaks under the influence of false conceptions." We would refer, on this point, to the argument, especially that advanced by Abercrombie.

Lorry has related very interesting cases, analogous to the hallucination of Tasso. The account of Madam Plautan's operation, during a state of magnetic ecstasy, is fully recorded, p. 316, with the examination of her body. Many of these stories are very curious, yet easily explicable, but others, for example the 108th case, are so shallow as to be worthless; yet we read some long arguments of our author in illustration of them. The passage nearest to our own views is this:—"The phenomena of clairvoyance, prevision, and second sight, depend on a sudden illumination of the cerebral organ, which lights up sensations hitherto in obscurity." This is figurative, but the meaning is correct. The 13th chapter is replete with proofs of the influence of the blood in the causation of hallucination. The case quoted from W. Hibbert, p. 329, is a good illustration, and a host of others are now passing through our mind. In fevers, often, the first symptom is illusion. With all these *facts,* we must still confess, with our author, that "it is probable that the production of hallucination is here owing to a morbid action of the nervous system and the circulation in the brain, but the *mode* of action is beyond our recognition."

In discussing the etiology of hallucination, M. de Boismont refers to the two elements of which an idea is composed. We hope we are correct in our interpretation, but psychological terms are unhappily so often *convertible,* that we almost fear to comment on them. We have believed that the definition of an idea was memory of impression on a sense, a spectre being an *intense* idea. This is, probably, the "signe sensible" of our author—the "partie somatique,"—his second element being the "partie psychique," or the "conception pure;" which may mean mere thought or clear *con*ception; but if this imply the notion of *innate* idea, of course we differ.

M. de Boismont here again objects to the notion of Ferriar and Hibbert, as to hallucination being essentially a symptom of insanity. That some people have visions there is no doubt; but we believe this phantasy, when in excess, ends in disorder—"maladie mentale;" perhaps, therefore, it is often a question of *degree* rather than of kind.

M. de Boismont naturally divides the causes of hallucination into moral and physical.

No doubt example—suasion—imitation—may seem to constitute the epidemic rationale; but this prevalence may sometimes depend on physical causes, such as the influence of atmosphere, &c. Now, we have yet to be convinced of our error in supposing that the state of the brain, the proximate cause, is not in all cases changed. In prevention, moral influence is often most powerful—education—early impression, as well as moral suasion,—nay, even the shafts of ridicule, may often check a spreading monomania.

Solitary confinement, especially in gloom or darkness, must be followed by phantasy, as the mind is completely thrown back and concentrated on itself. Anecdotes illustrative of this might be cited; but they, as well as the anecdotes of Silvio Pellico—the stories in Scott, Byron, &c. &c. are already well known. We have, p. 369, very interesting allusion to the influence of surrounding events in imparting *character* to illusions. In the two hospitals of which our author was director, we might observe two grand classes—the old inmates, "ancienne societé," were admitted before 1793, during the era of agitation and terror; *their* phantasy was demonomania. The second set did not arrive until the new order of things was established; *they* were the subjects of cheromania.

Then come we to the influence of fanaticism, or false religion.

"The religion of the ancients, which peopled the whole universe with divinities or genii (demons, &c.), led naturally to belief in the power and materiality of spirits. The influence which the doctrines of Plato (probably borrowed from Zoroaster), imparted to the subject, was immense."

"This *direct* intervention of the evil spirit in human affairs, at once recognised and generally admitted, was the inevitable cause of all sorts of follies and extravagancies."

We might allude, in illustration, to the errors of Sir Matthew Hale, Sir Thomas Brown, the fate of Spinello, &c. &c., but we have neither space nor time.

The diabolical epidemic of 1459 in Arras, and the religious possession of Loudon, were the immediate effect of imitation. The influence of magic, however, seems to have begun to be of greater effect about 1484, after the issuing of the bull of Innocent VIII.

At the end of the sixteenth century came out two arch visionaries, Dee and Napier. Of all these, our author gives us most circumstantial relations.

The conversion of humans into brutes was believed by Ulysses, Herodotus, and even St. Augustin. From Wierius we have learned the demonomania of Besançon in 1521, in which men professed to be "loups-garoux," and pleaded guilty to the killing and eating of young girls. The stories of Miss Lee, and Sir George Villiers are cited, to which we allude, that we may quote a portion of our author's conclusion in reference to these facts. It is certain that a great number of apparitions take place without reference to any important or remarkable event, others "per hasard se sont realitées."

The cases of Colonel Gardner (Hibbert), and of Lord Herbert of Cherbury, are quoted as contrasts of devout or celestial and demoniac influence. Gardner was converted, and Lord Herbert, believing he had divine authority, published his infidel book.

The cases of Charles IX., and of Jervas Matcham, and of Beaufort, quoted from Dr. Winslow's work, are re-excited impressions, or memory of crime; and the well known story of Tasso's familiar spirit, concludes the moral causes of hallucination.

The physical causes of hallucination are divided into three sections. In the first, we have hereditary diathesis—sex and climate; but climate should not be associated with temperament.

Hereditary tendency may probably depend on nervous or vascular influence.

Regarding the sexes, of 136 patients in the author's establishment, 63 were males, 73 females. Hallucination may appear as early as the seventh year. The author relates an interesting story of a girl of twelve years of age, who, in her ecstasy, had angelic visions. "See ye not," she would say, "those angels in heaven? they are crowned with flowers; they are coming this way to seek me." During the paroxysm her pulse was scarcely perceptible; her skin was icy cold, and her countenance ashy pale.

It is recorded, that among the Cevennes and the predicants of Sweden, children of even five years of age were discovered.

Among temperaments, the bilious or melancholic will, from its accumulations of dark blood, be most predisposed to hallucination; and, we know, intense thought will favour this; as was probably the case with Socrates and Plato, and the host of poets and other abstract and deep thinkers.

We think too much stress is laid on the influence of the arctic climate. True, the Laplander, the Ostiac, the Samoiede, are all subjects of illusion, but they are grossly ignorant; they cannot reason on the grandeur and sublimity of the natural phenomena around them—reason fails, and wild imagination takes its place—but the same is observed among the dwellers in the Hartz, in Switzerland, and in the gorges of the Himalaya and the Ghauts; *warm* as well as cold mountaineers are equally visionary and superstitious. Thus the Chaldean became a shrewd astrologer.

So hallucinations take on a stamp from surrounding circumstances. Those "of the city are constantly distinguished from those of the country by very perceptible shades." Of this, the Bar-gheist, the phoca, the Bodach-glas, the fetch, the wraith, may form apt illustrations, as well as the mirage, the calenture, the Schattenmau, &c., &c.

In the *Gazette de Mons*, we learn that Dr. Boismont, the companion of our aeronaut, Green, is cited as having recorded very curious atmospheric phenomena. The second division is chiefly confined to the influence of alcoholismus and narcotism. It is to be regretted that we have so much opium eating in England, but we should *not* be classed with the Eastern voluptuaries, of whom Moqueville has given so debasing an account. "Their infatuation is such that the certainty of death, with its direful forerunners, cannot deter them from swallowing the funereal poison." A deplorable case is recorded of one who, for four consecutive hours, took one ounce of solid opium per hour!

In illustrating the effects of hachish, the author alludes to the cases of three young merchants of Marseilles; these, however, are more physical than psychical, and might have been omitted. The mental effects of stramonium are somewhat similar to those of hachish. The third section is composed of forms of illusion complicated with the vesaniæ. The fourth of those associated with the tremores, as catalepsy, nightmare, &c., &c.; and the fifth, of combinations of illusion with cerebral congestive, and inflammatory maladies. We have merely time to commend but not to review them.

We come now to a question of much depth and importance—"hallucination studied in reference to psychology, history, and religion." We have so often alluded to the hair line of demarcation between sanity and derangement, that our own words almost ring in our ears. These are the words of the author:—"Who can say, 'here ends reason, here begins insanity?' What naturalist can define the line of demarcation between the animal and vegetable kingdoms—thus is it with our present subject."

Now, we believe, that monomaniacs and lunatics may often produce great works on the principle of concentration; but it does not follow, as we are told, that Alexander and Columbus were mad in the true sense of the word. M. de Boismont offers some sensible remarks on the nature of cerebral impression, as well as on the power of second sight, and of "calling," like Owain Glyndwr, "spirits from the vasty deep," and those phantoms which come without calling, the ghosts of memory and those of recollection.

The author's remarks on the combination of mind and matter, as well as those on the antipodean causes of illusion, will be read with much interest. Although somewhat tautological, we do not think they have been elsewhere better treated.

In the following passage the author more openly expresses his belief in the *innateness* of idea. Ideas "may be referred to two sources, those which owe their materials to the senses (idées sensuelles secondaires), and those which have their origin in the soul—from God, (idées spirituelles primitives)." Therefore he takes with great reservation, the "nihil in intellectu quod non prius in sensu." We may merely observe, that what the author terms *general* ideas, as of quality, existence, &c., are, properly speaking, *notions*. The association of ideas, as a cause of illusion, is a subject of much interest, especially in a *remedial* point of view. To prove this we are favoured with the so often quoted "*Eagle's-nest anecdote*" of Dr. Rush.

It is clear that Mons. de Boismont considers the intellect, or the mind, as an *abstraction;* and, we fear, that with this metaphysical tendency, his attention might be too much diverted from the brain, which, if he allows it to be the organ or habitat of mind, he considers at most but *unity*, and not as a duality or plurality of organs. We write this after reading the following passage, which we venture to quote in the original language.

"Il serait bien plus étonnant qu'avec des sensations différentes de celles qu'éprouvent les hommes en santé qui l'environnent, le malade continuât de raisonner comme eux; c'est alors véritablement que la raison serait pervertie et bizarre.—Parceque le cerveau peut être la cause du délire, gardons nous de conclure que c'est le cerveau qui pense et qui raisonne ; ce serait dire que l'œil disserte sur les couleurs parce qu'il nous les fait distinguer avec plus ou moins de vérité."

The author's mode of reproduction of ideas, that is, the theory of memory, coincides with our own notions.

These, with the remarks of the author of Gall and of Crichton, on the seemingly special accumulation of ideas previous to dissolution, are referred to. It is a subject of deep and almost awful interest, involving the transit of the immortal spirit. It has been elsewhere fully discussed.

When our learned author objects to M. Baillarger for affirming that hallucination is favoured by *involuntary* memory and imagination, and he himself tells us that the exercise of attention is the cause of illusion in many cases, they are both right, but each looks on one side of the shield only. What matters it, for the sake of an argument, by what means, or exciting causes, cerebral hyperæmia is induced, when such is the effect? Whether memory, excited in a congested brain without the will, or by voluntary and strained attention, as in the adduced case of Sir Joshua Reynolds? The difference is this, the involuntary phantasy will usually be velut ægri somnia *vanæ,* the voluntary more rational, and perhaps even intellectual.

Of the phantasy springing from devotion to an absorbing subject, we translate the following interesting case, which is new to us:—"A young man was deeply engaged in the projection of a canal scheme. One day, after his attention had been devoted to the subject, he marked on a geographical chart the line of a canal which was to be made in that district. Suddenly he saw a yellow-covered brochure with this title, 'Project for Opening a Canal to the Plains of Solòque.' After awhile, the fantastic brochure disappeared."

Although we differ, as well as M. de Boismont, from Reid, &c., we do not coincide with him in this rather transcendental sentence:—"The first origin of hallucination ought therefore to be sought for in the oblivion of the two grand laws which should govern mankind, the knowledge of God and of himself."

The two natural orders into which M. de Boismont divides hallucinations are simply, the illusions of a multitude (epidemic), and those of the individual. Of the first, he is satisfied with the history of the one grand project of Peter the Hermit. And these are two of the visions of the army:—"At the battle of Doryglée they saw St. George and St. Demetrius fighting in their ranks." "In the centre of the melée at Antioch, a celestial phalanx, fully armed, came down from heaven, led by the martyrs St. George, St. Demetrius, and St. Theodore."

Of the second order, we have many interesting histories; among them the visions of Loyola, and Joan of Arc, and Luther,—all of whom would probably be termed monomaniacal by their enemies; the unchristian creed of the Jesuits—the *unsexing* of the Maid of Orleans and the conference of Luther with the devil, prove their phantasy, at least on certain points. Had Joan of Arc been beaten in her first encounter, she would have been a madwoman or a traitress; but we admire, with Ideler, the pretty *novellette* which M. de Boismont has made of her. And had George Fox, the founder of Quakerism, done nothing else than buried himself in an old tree as he did, and seen celestial visions, would not he have been set down a lunatic?

With all his leaning toward the *physiology* of hallucination, our author yet comes very close to our opinion in this brief sentence:—"We have already said, elsewhere, that simple (pure) hallucination without complication with one form of insanity, seems to us as rare as cases of true monomania." We are treated, page 521, with a sly lunge at Arnold and Hibbert, who, with all rational psychologists, believe that the days of special inspiration are past:—"Il ne faut pas oublier qu'Arnold et Hibbert sont *Protestants*."

In the sixteenth chapter we are met by a very subtle question: "Is hallucination psychical, or psycho-sensorial; in other words, is it purely intellectual, or needs it the intervention of a sense?" And the opinions of Baillarger, Burdach, Bayle, Bostock, Müller, are all well analyzed on this point. That not only sense, but sensation, may be revived by memory, the ghost of the amputated limb prominently proves the explanation is psycho-sensorial. But where is the evidence or proof of a purely psychical illusion, which may not be traced to something once heard, seen, or read of? Of course we think the case of Dr. Bostock himself, who affirmed that the "images followed the direction of his eyes," tends to prove too much and weaken the argument. His case was purely optical and not psychical. We see clearly a fallacy in the reports of Nicolai, Bostock, and Cardan, but must not pause to refute them.

The very curious cases of "Hallucination Dédoublées" have been so ingeniously explained by Wigan, that we must refer the reader to his work on duality of mind, if he wishes to dip deeper into psychical mystery.

One of the most interesting subjects to the psychologists is that species of hallucination which *impels to action*: this is the illusion so dangerous to society. It is true, that it sometimes excites to good actions, but we fear the balance is woefully in favour of the malignant spirit.

When in the silence of night we hear the cry of a lunatic, we may rest assured, without fear of contradiction, that his agitation is caused by hallucination.

The calculation which M. de Boismont has made on the comparative frequency of night and day illusions, is as follows :—

"Of 144 cases of hallucination,

| | |
|---|---|
| During the night . . . . . . . . . . | 62 |
| During the day . . . . . . . . . . . . | 50 |
| During night and day . . . . . . . . . | 32" |

M. Baillarger has presented to the Academie Royale de Médicine a more elaborate report, to which we refer with confidence.—(p. 569.)

We have often been struck with the fact to which the following sentence refers :—"The smell, like the other senses, is one cause of illusion in the insane : a rose exhales a sulphitic odour ; many discern a taint in their food, arising from faults in their digestion, or from a parched condition of the mucous membrane of the mouth; and they hence reject their diet, under the apprehension that it contains poison."

We were surprised to perceive that the hallucinations of children are adduced as arguments against the notion of cerebral lesion. But the development within the infantile cranium, renders it always prone to great changes in the circulation ; and it will be remembered that it is during the recumbent position, when the head is low, that the illusions of children almost invariably supervene, and not while they are standing or sitting.

Periodicity, or remission of symptoms, it may be remarked, does not disprove organic lesion. We have repeatedly seen this occur in cerebral tubercle and effusion.

The duration of a paroxysm, especially when dependent on hyperæmia, may be very brief—a few hours only—vanishing when the circulation is restored.

M. de Boismont has seen hallucination last twenty years in the insane.

Dr. Marc has noticed that the more unusual and eccentric the actions of the insane, the more we may presume that the cause of them is illusion ; and we may be sure when the talk of the lunatic is of angels and demons, that he is then in a state of phantasy. In page 595, we have some very correct remarks on diagnosis, well worth perusal.

On the subject of treatment, Leuret seems to have been the first systematic writer. We quite agreewith our author in this sentence,

"Etiology, symptomatology, and clinical observations, show the importance of physical and moral remedy—alone or combined—in the treatment of hallucination."

The révulsion morale of M. Leuret, we are certain, will often fail, if uncombined; a healthy state of the organ must be ensured, the good seed will grow up in it.

The medico-legal consideration of hallucination is of deep interest in the study of psychology.

The danger to society with which phantasy is fraught, is especially increased when the visions of a lunatic are both subjective and objective. If it were merely the hallucination that a man's enemies were following him, he would probably seek his safety in flight; but if, at the same time, he converted natural objects into phantoms, if he believed that his attendants, or his physician, *were those very enemies,* murder might often be the climax of the vision. It is, therefore, when hallucination and illusion are combined in the same patient, that the greatest care and watchfulness should be enjoined; and this, even when the motives of the unhappy being are what we might term pseudo-holy. Of this state, a very painfully interesting history is given in page 657. How far Ravaillac, Clement, and Jonathan Martin, were influenced by this illusion, we have not been able to conclude.

The medico-legal chapter of M. de Boismont's work is a complete romance; for, although it is replete with sound reasoning, it is profusely embellished by the most interesting novellettes. We think that many of the stories might have been omitted, for conciseness is the quality we most prize in a book on this subject. The volume displays, however, so much research, and bears so high a stamp of classicality about it, that all must receive instruction from its perusal.

We must confess, in conclusion, that the morbid anatomy, the proximate cause, the psycho-pathology, of hallucination, is still sub umbra; and we may, perhaps, be long foiled in unravelling the mystery. We feel much pleasure in commending this able and philosophical work to the patient study of all psychologists.

